# Small Thanks, Big Impact: The GREATIX Effect in a Psychiatry of Intellectual Disability Hospital

**DOI:** 10.1192/bjo.2026.11412

**Published:** 2026-06-30

**Authors:** Patrick Kielt, Kirsty Duddy, Salman Buttar, Maame Afua Gbolooteye

**Affiliations:** NIMDTA, Belfast, United Kingdom

## Abstract

**Aims:**

This quality improvement project aimed to enhance staff morale within the Psychiatry of Intellectual Disability service at Lakeview Hospital, part of the Western Health and Social Care Trust. This was achieved through the development and implementation of a ‘GREATIX’ scheme designed to recognise and celebrate exemplary staff practice between April 2025 and June 2025.

**Methods:**

Baseline staff morale was assessed using a Likert-style questionnaire distributed to the multidisciplinary team working within Lakeview Hospital. Following this, a ‘GREATIX’ nomination process was introduced, allowing staff to nominate colleagues for recognition. Fortnightly meetings were held with charge nurses to review nominations and determine successful recipients. Eligibility required nominees to demonstrate behaviours aligned with the Trust’s core values: working together, excellence, openness, honesty, and compassion. Successful nominees were awarded a ‘GREATIX’ certificate and a £5 voucher. In July 2025, following implementation of the intervention, a repeat Likert-style survey was circulated to reassess staff morale.

**Results:**

The baseline survey received 28 responses, while the post-intervention survey received 22 responses. Mean self-reported tiredness following a shift decreased from 3.71 atbaseline to 2.41 post-intervention (Likert scale: 1=not tired, 5=extremely tired). Perceived impact of work showed an improvement, with mean scores increasing from 3.54 at baseline to 3.73 post-intervention (Likert scale: 1=little difference, 5=huge difference). Feelings of collegial support also increased, with mean scores rising from 3.68 to 3.87 (Likert scale: 1=not supported, 5=well supported).

Both surveys included free-text responses regarding ward morale. Baseline responses described morale as “OK”, “pretty good”, and “adequate”. Post-intervention responses suggested a modest improvement, with comments such as “a little better” and one respondent describing the GREATIX scheme as “a lovely addition”.

**Conclusion:**

Implementation of the GREATIX scheme was associated with improvements in staff-reported energy levels after shifts, perceived collegial support, and feelings of having made a positive contribution at work. These findings suggest that structured recognition initiatives may have a positive impact on staff morale. Further PDSA cycles are planned within the Western Health and Social Care Trust to build on these outcomes.

